# Sex Differences in Mathematics and Reading Achievement Are Inversely Related: Within- and Across-Nation Assessment of 10 Years of PISA Data

**DOI:** 10.1371/journal.pone.0057988

**Published:** 2013-03-13

**Authors:** Gijsbert Stoet, David C. Geary

**Affiliations:** 1 Institute of Psychological Sciences, University of Leeds, Leeds, United Kingdom; 2 Department of Psychological Sciences, Interdisciplinary Neuroscience Program, University of Missouri, Columbia, Missouri, United States of America; Tel Aviv University, Israel

## Abstract

We analyzed one decade of data collected by the Programme for International Student Assessment (PISA), including the mathematics and reading performance of nearly 1.5 million 15 year olds in 75 countries. Across nations, boys scored higher than girls in mathematics, but lower than girls in reading. The sex difference in reading was three times as large as in mathematics. There was considerable variation in the extent of the sex differences between nations. There are countries without a sex difference in mathematics performance, and in some countries girls scored higher than boys. Boys scored lower in reading in all nations in all four PISA assessments (2000, 2003, 2006, 2009). Contrary to several previous studies, we found no evidence that the sex differences were related to nations’ gender equality indicators. Further, paradoxically, sex differences in mathematics were consistently and strongly inversely correlated with sex differences in reading: Countries with a smaller sex difference in mathematics had a larger sex difference in reading and vice versa. We demonstrate that this was not merely a between-nation, but also a within-nation effect. This effect is related to relative changes in these sex differences across the performance continuum: We did not find a sex difference in mathematics among the lowest performing students, but this is where the sex difference in reading was largest. In contrast, the sex difference in mathematics was largest among the higher performing students, and this is where the sex difference in reading was smallest. The implication is that if policy makers decide that changes in these sex differences are desired, different approaches will be needed to achieve this for reading and mathematics. Interventions that focus on high-achieving girls in mathematics and on low achieving boys in reading are likely to yield the strongest educational benefits.

## Introduction

In recent decades, women’s participation in the workforce and pursuit of higher education has increased substantially, but there continue to be striking sex differences in college majors and career choices. Sex differences are particularly notable at the highest levels of scientific achievement; for example, under 3% of Nobel laureates in science are women, and no women have so far received one of the top three awards in mathematics (the Fields Medal, the Abel Prize, and the Wolf Prize).

A much publicized study showing that boys greatly outperform girls at the highest ranges of mathematics ability [1] ignited the debate about underrepresentation of women in Science, Technology, Engineering, and Mathematics (STEM) fields in the early 1980s. In a talent search among students in secondary education, researchers reported a 13∶1 ratio of high-achieving adolescent boys to girls in the U.S. [1]–[2]. For high-achieving U.S. adolescents, the ratio dropped to about 4∶1 by the mid 1990s and has been stable since that time [3].

The causes of the sex difference in mathematics performance, in general, have been extensively discussed over the ensuing years. A number of biological [4] and socio-cultural causes [5]–[6] have been proposed, as well as debated [7]–[9]. There is, however, little doubt that both nature and nurture play a role (for extensive reviews and theoretical proposals see [10]–[11]). The multicausal background of the sex difference in mathematics holds true for other cognitive sex differences as well, in particular reading, in which girls typically do better. More complex, though, is the international variation in the pattern of these sex differences.

A number of scholars have argued that the international variation in the sex difference in mathematics performance is correlated with country’s implementation of gender-equality measures [12]–[14], but there is no concensus on this matter. Researchers who analyzed the PISA data from 2000 cautioned against overinterpreting the positive weak correlation observed [15], and a more recent study did not find such a correlation in the 2007 TIMMS and 2009 PISA data, but nevertheless suggested that girls benefit from equality measures [16]. Kane and Mertz [16] attributed the lack of a correlation between the sex differences in mathematics and equality policies to the fact that not only girls’ performance is higher in countries that have good equality measures in place, but that boys benefit as well. In other words, the increase in both boys’ and girls’ performance means not only that the there is overall improvement, but that the sex differences will be maintained. This ongoing debate not only relates to between-nation variation in sex differences, but also to within-nation differences. A recent analysis of a sample of 20,000 U.S. children found no evidence that the sex difference in mathematics performance is related to negative socio-cultural factors (e.g., low parental expectations or biased tests), and that the sex difference is in fact particularly large among children in environments that are potentially beneficial to cognitive and academic development [17]. Thus, at this time, there is no consensus regarding the effect of formal and informal practices that promote gender equality on girls’ and women’s STEM achievement.

In the current paper, we focus on two related issues in regard to the sex differences in mathematics and reading performance: 1) We explore further the paradoxical relation between sex differences in mathematics and sex differences in reading performance. 2) We explore further whether sex differences in reading and mathematics are related to national indicators of gender equality.

We analyzed the sex differences in all four available assessments (carried out in 2000, 2003, 2006, and 2009) of the Programme for International Student Assessment (PISA), funded by the Organisation for Economic Co-Operation and Development (OECD, http://www.oecd.org). It is the largest multi-national standardized assessment of academic achievement of 15-year olds, the oldest age for which schooling is mandatory in many member nations. Nearly 1.5 million students from 75 different countries or economic regions participated (see Materials and methods). It is ideal for studying cross-cultural comparisons of sex differences in scholastic achievement, because the content of the tests is the same for all countries, and focuses on measuring the problem-solving skills of students in different domains (mathematics, reading, science) rather than on specific curricula (see Materials and methods).

Another strength of these data is that PISA scores are strongly correlated with the prosperity of nations, which indicates that the competencies measured have real-life validity [18]. We extend previous analyses [13]–[15] of sex differences in mathematics achievement in single-year PISA assessments to all four years, and more critically place these differences in the context of a hitherto unexplained paradoxical finding: The smaller the sex differences in mathematics, the larger the sex differences in reading (i.e., countries with a smaller sex difference in mathematics have a larger sex differences in reading, and countries with larger sex differences in mathematics a have smaller sex difference in reading).

This inverse relation between the sex differences in mathematics and reading achievement poses a critical challenge for educators and policy makers who might wish to eliminate such differences. After all, as we demonstrate, it means that that there are currently no countries that have successfully eliminated *both* the sex difference in mathematics (i.e., girls typically scoring lower than boys) *and* the sex difference in reading (i.e., boys typically scoring lower than girls). There are different possible explanations of this relation. One possibility is that when girls’ overall academic performance improves in a country, they reduce the sex difference in mathematics and increase their advantage in reading [13] [15]. It is also possible that there is a trade-off between mathematics and reading skills due to limited resources. Resource limitations can, in turn, have different causes. They can be related to economic resources of nations making a trade-off between investing in reading or mathematics education, or they can be related to the time spent within curricula on either reading or mathematics. A third possibility is that there are sex differences in the sensitivity to general living conditions, including the quality of educational environments (such sensitivities might be biological in origin, see pp.291–294, pp.411–412 of [10]). At this point, we have no definite answer to what can explain the correlation, which means that it requires further study. We argue that our in depth analysis of all four PISA assessments can exclude the first hypothesis.

## Results

Before detailing the relation between the sex differences in mathematics and reading, we examine each separately.

For all analyses, we express sex differences in PISA score points. These scores are not “raw” scores, but result from a statistical analysis that normalizes student scores (see Materials and methods) such that the average student score of OECD countries is 500 points with a standard deviation of 100 points. The advantage of this is that scores become easily comparable and differences easily to interpret. For example, a 10 point difference between boys and girls reflects approximately 1/10th of a standard deviation.

### Sex Differences in Mathematics Performance

**Figure 1:**
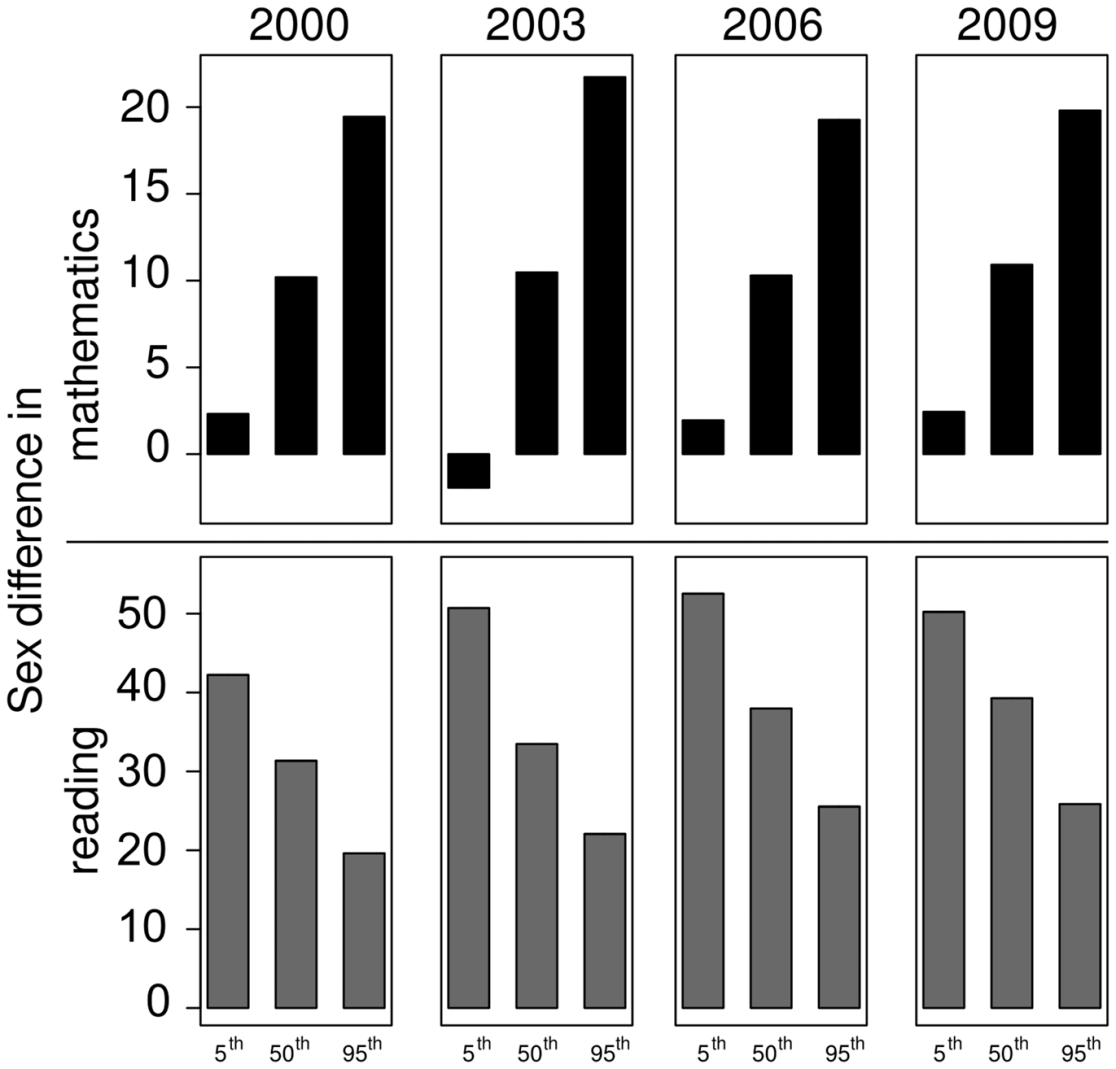
Sex differences in mathematics (top) and reading performance (bottom). Top: For each PISA assessment, the sex differences in mathematics (boys’ performance – girls’ performance) is displayed for the 5th, 50th, and 95th percentile of the performance distribution. Bottom: Similar for sex differences in reading (girls’ performance – boys’ performance).

Across nations, the mean overall sex difference in mathematics was small but remained relatively stable over the ten years at 10 to 11 points (Figure 1, top). The difference was practically non-existent among the students at the bottom of the mathematics performance continuum, but it was larger at increasing performance levels. Comparing the bottom 5% of boys (relative to all other boys) to the bottom 5% of girls (relative to all other girls), the difference in mathematics achievement ranged from a 1.9 point difference (2003), favoring girls, to a 2.4 point difference (2006), favoring boys. In contrast, boys scored from 19.3 (2006) to 21.7 points (2003) higher than girls at the high end of performance.

Another common way of expressing the sex difference is the ratio of boys to girls at different points along the performance distribution (Table 1). Of particular interest are the ratios at the high end of mathematics performance, where the largest sex difference is traditionally reported [1], and which contribute to an overrepresentation of men in STEM fields. This is because the students who are most likely to enroll in a STEM field in higher education are high achievers in secondary education [19]. For the 33 countries that participated in all four of the PISA assessments (i.e., 2000, 2003, 2006, and 2009), a ratio of 1.7–1.9∶1 was found for students achieving above the 95th percentile, and a 2.3–2.7∶1 ratio for students scoring above the 99th percentile. The data for *all* participating countries showed a similar pattern (Table S2).

**Table 1:** Ratio of boys to girls in mathematics achievement at various percentiles.

Achievement Percentile	2000	2003	2006	2009
1^st^	1.1	1.3	1.1	1.0
5^th^	1.0	1.1	1.0	1.0
95^th^	1.8	1.9	1.7	1.7
99^th^	2.7	2.3	2.3	2.3

### Sex Differences in Reading Performance

In contrast to the sex difference in mathematics, the difference in reading, favoring girls, receives relatively little attention, despite the fact that the average sex difference in reading was three times larger than the sex difference in mathematics (Figure 1, Table 2). Not only was the sex difference in reading relatively large, the overall average difference increased from 32.0 points in 2000 to 38.8 points in 2009, *t*(32) = −6.25, *p*<.001.

**Table 2:** Ratio of boys to girls in reading achievement at various percentiles.

Achievement Percentile	2000	2003	2006	2009
1^st^	3.1	4.1	4.8	5.9
5^th^	2.5	2.8	2.9	3.2
95^th^	0.6	0.6	0.5	0.5
99^th^	0.5	0.5	0.4	0.4

Further, the very poor performance of boys at the low end of reading achievement drove, in large part, the overall sex difference and the increase in it (Figure 1, bottom). In the 2009 PISA, the bottom 5% of boys in reading skills scored 50 points lower than the bottom 5% of girls. In effect, this means that boys at the 13th percentile of the boys’ reading distribution scored at the same level as girls at the 5th percentile of the girls’ distribution. Girls had higher reading scores at the 95th percentile as well, but here the sex difference was only about half that found at the bottom.

At the first percentile of reading performance, the ratio of boys to girls ranged from 3.1∶1 in 2000 to 5.9∶1 in 2009, and at the fifth percentile from 2.5∶1 to 3.2∶1 (Table 2). Similarly, at the high end, there were fewer boys than girls, but the difference was less extreme (at the 99th percentile 0.4–0.5∶1). The data for *all* participating countries showed a similar pattern (Table S3).

### The Relation between the Sex Differences in Mathematics and Reading Performance

Previously, the relation between the sex differences in reading and mathematics were noted by Marks [15] for the first PISA assessment and by Guiso and colleagues [13] for the second assessment. We extend and elaborate on this relation and show that the sex differences are indeed systematically and inversely related in all four PISA assessments between- and, critically, within-nations.

**Figure 2:**
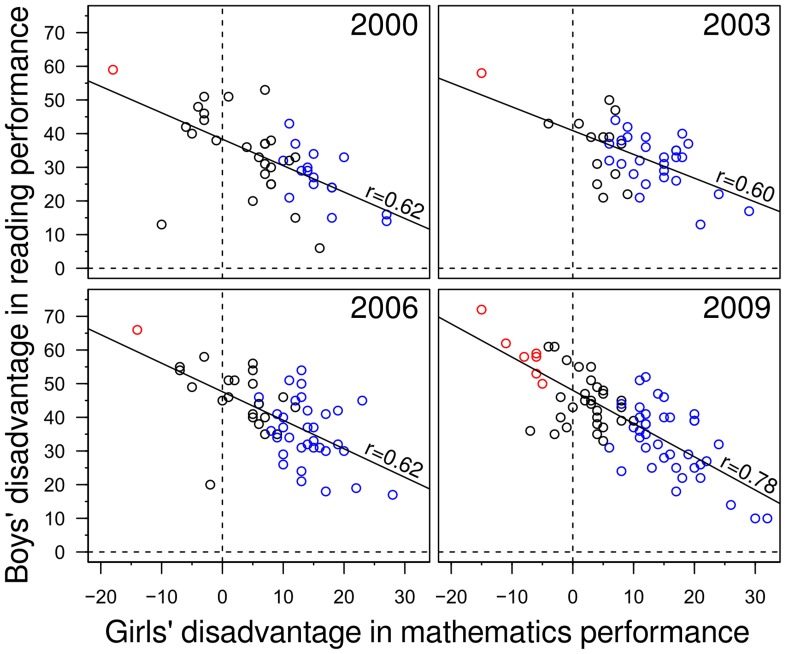
Negative correlation between boys’ disadvantage in reading achievement (y-axis) and girls’ disadvantage in mathematics achievement. Each data point indicates the sex differences of one country. Positive values indicate a larger disadvantage, negative values an advantage. Red points indicate nations in which girls’ mathematics achievement is significantly higher than that of boys; blue points indicate nations in which boys’ mathematics achievement is significantly higher than that of girls; and, black points indicate nations in which there is no statistically significant difference in mathematics achievement. The advantage of girls in reading achievement is statistically significant in all nations, except for 2 in 2000 (Israel, Peru) and one in 2003 (Liechtenstein).

**Figure 3:**
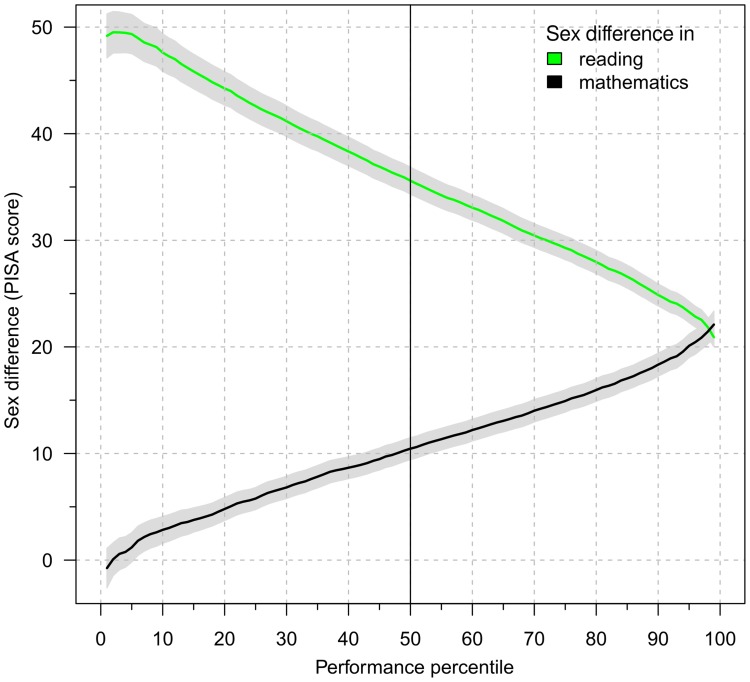
The magnitude of the within-country mathematics and reading sex differences across performance. The two curves represent the magnitude of sex difference in mathematics (black) favoring boys and the sex difference in reading (green) favoring girls in all 33 countries that participated in all four PISA assessments (2000,2003,2006 and 2009). Grey shading indicates ±1 SEM. Within these countries boys at the 50th percentile of the distribution of boys’ scores have a 10 point advantage in mathematics over girls at the 50th percentile of the distribution of girls’ scores. There is no gap for the lowest performing students and a doubling of the average gap for the highest performing students. For students at the 50th percentile, girls reading advantage is about 37 points and increases for lower performing students and decreases for higher performing students. The relation between the two gaps within these countries is the same as found between countries (Figure 2). For percentiles with a small mathematics gap, the sex difference in reading is large. The larger the sex difference in mathematics within these countries, the smaller the reading gap.

We found that the across-nation inverse correlations between the sex differences in reading and mathematics were consistent and strong in all four assessments (Pearson’s *r* ranging from −0.60 to −0.78, *ps* <.001, Figure 2). But the inverse relation between the sex differences in mathematics and reading was also found within countries along the performance continuum (Figure 3, see Material and methods for how the curves are determined). At the highest performance level the sex difference in mathematics was largest and the sex difference in reading smallest.

### Gender Equality and Sex Differences in Achievement

Multiple research teams have studied the relation between sex differences in mathematics on the one hand and national gender equality, economic, and human development indicators on the other hand [13]–[15] [20]. The gist is that indicators of gender equality are positively correlated with girls’ mathematics achievement. We found that mathematics and reading performance of *both* girls and boys correlated positively with living standards (Human Development Index, HDI), which itself correlated positively with various gender-equality measures, such as the Gender Empowerment Measure (GEM) or the Global Gender Gap Index (GGGI, Table S4). But across the decade, we found no *consistent* correlations between the sex differences in mathematics or reading and these variables (Table 3).

**Table 3:** Correlations between sex differences in mathematics and reading on the one hand and human development and equality indicators on the other hand.

	Sex difference in mathematics				Sex difference in reading			
	2000	2003	2006	2009	2000	2003	2006	2009
HDI	0.36*	0.01	0.24	0.24	0.02	0.25	−0.04	0.09
GII	−0.22	0.01	−0.16	−0.12	−0.27	−0.28	0.06	−0.14
GDI	0.34*	−0.01	0.23	0.21	0.05	0.26	−0.03	0.13
GEM	0.11	−0.21	0.03	0.24	0.31	0.33	0.09	−0.03
GGGI	−0.10	−0.42**	0.04	0.12	0.38*	0.50 **	−0.01	0.05
Gini	0.17	0.05	0.18	0.25	−0.39*	−0.16	−0.20	−0.49***

HDI: Human Development Index. GII = Gender Inequality Index. GDI = Gender Development Index. GEM = Gender Empowerment Measure. GGGI = Global Gender Gap Index. Gini = Gini coeffient. * *p*<.05; ** *p*<.01; *p*<.001.

**Figure 4:**
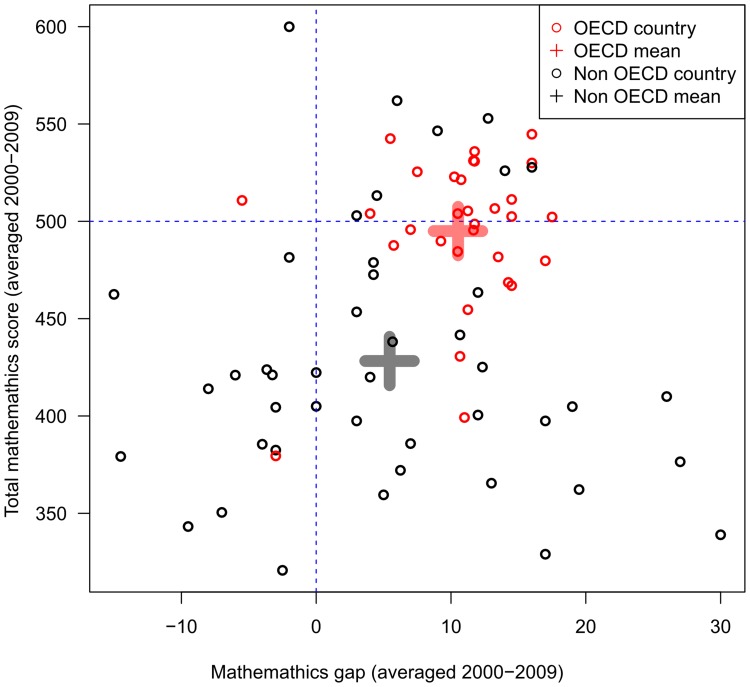
Relation between sex difference in mathematics and overall mathematics score for OECD and non-OECD countries. The mathematics scores have been averaged for the four assessments, which means that some countries’ scores are based on four assessments (e.g., Germany and 32 other countries/regions which participated in all four assessments), and some countries’ scores on only one assessment (e.g., Malta and 15 other countries). Sex difference in mathematics equals boys’ mean score - girls’ mean score. The OECD countries not only have higher overall scores, their mathematics gap, favoring boys, is more tightly clustered between −5.5 and 17.5 points (*M* = 10.5,*SD* = 5.1). The two outliers are Iceland (HDI rank = 2, GGGI rank = 1) and Georgia (HDI rank = 61, GGGI rank = 40). In contrast, there is considerable variability in the non-OECD countries (between −15.0 and 30.0 points, *M = *5.4, *SD* = 10.5), with boys’ having higher mathematics achievement in some of them (e.g., Costa Rica) and girls having higher mathematics achievement in others (e.g., Albania). The same analyses applied to the 4 individual PISA assessments show the same pattern.

If anything, economically developed countries with strong gender-equality and human development scores tended to have a larger sex difference in mathematics than less economically developed countries (e.g., for non-OECD countries the sex difference in mathematics was 5.4 vs 10.5 points for OECD countries, *t*(73) = −2.5, *p* = .02, Figure 4). Further, we found considerable variation among lower scoring countries, with some showing a large sex difference in mathematics achievement favoring boys and others favoring girls (Figure 4, Table 4). In other words, the sex differences in mathematics was more consistently found among higher-achieving nations, a pattern which coincides with the larger sex difference in mathematics in high-achieving students (Figure 3).

**Table 4:** Percentage of countries with a sex difference in mathematics >0.

	Percentage of countries in which boys score higher than girls in mathematics.	
	Of countries with average mathematics score of > = 500	Of countries with average mathematics score <500
2000	88.9%	70.8%
2003	95.2%	95%
2006	90.9%	85.3%
2009	95.0%	72.2%

The average PISA score of all students in OECD countries is 500. A high percentage of the countries that have an overall mathematics score over 500 in mathematics have a sex difference in mathematics greater than zero (no country had a score of exactly 500). This occurs less frequently among the lower scoring countries, although the difference was negligible in 2003. Altogether, this shows that boys exceeding girls is more strongly associated with an overall high mathematics performance.

## Discussion

We found that the paradoxical relation between the sex differences in mathematics and reading across different nations occurred in each of the four PISA assessments carried out over 10 years. That is, countries with a smaller sex difference in mathematics tended to have a larger sex difference in reading. This inverse relation between the sex differences in mathematics and reading is not merely an effect that emerged between countries, it also occurred across the performance distributions within countries. The sex difference in mathematics was non-existent at the lower end of the performance distribution, but the sex difference in reading at the lower end was at its peak. As with the between-country findings, the larger the sex differences in mathematics within countries, the smaller the sex differences in reading.

This finding has important implications for the way we think about the nature of sex differences in mathematics and reading. Previously, Guiso and colleagues hypothesized that the negative correlation of mathematics and reading scores between countries might simply reflect that girls in countries with good resources will reap the benefits in both mathematics (improving in comparison to boys and thus reducing the sex difference) and in reading (improving in comparison to boys, and thus increasing the already existing sex difference) [13]. Our finding that the sex differences in mathematics and reading are inversely related, not only between but also *within* countries, is inconsistent with this hypothesis. The hypothesis relies on an assumption that is not directly testable with the data, namely that increased resources are associated with increased performance. Nevertheless, it is plausible at the national level and we make the same assumption. But if that assumption and the hypotheses are true, we would expect that the benefits to children from increased resources would be reflected in both mathematics and reading achievement; in other words, within countries, the sex differences in both mathematics and reading should follow a similar pattern, and not the opposite pattern (as in Figure 3).

The finding that the sex differences in mathematics achievement were larger at the high end of the continuum is important for understanding the underrepresentation of women in STEM fields. Whereas some have argued that the average sex difference in mathematics in the second PISA assessment is negligibly small [14], it is important to note that the students who will enroll in STEM fields in higher education will most likely come from the high-end of the performance continuum [19]. Therefore, it is not sufficient to only consider average performance levels (where the sex difference in mathematics achievement was 10–11 points) when considering the implications for STEM fields, but also to examine the sex difference at the high end (where it was around 20 points and the boy to girl ratio is 2.3 to 1, Table 1). We believe there is a link between the mathematics sex difference at the high end of performance in 15 year olds and the underrepresentation of female students enrolling in a mathematics degree program. To fully assess this hypothesis requires a separate investigation, but our preliminary analysis (for details, see Figure S1) of the enrollment in mathematics degree programs in two relatively egalitarian countries (United Kingdom and The Netherlands) for which we have the relevant data suggests that such a link indeed exists; not only has the proportion of male and female undergraduate students in mathematics remained stable since at least a decade, the ratio of men to women is similar to that observed in the high end of the mathematics performance in the PISA data.

In any case, the inverse relation between the sex differences in reading and mathematics, especially at the extreme ends of the achievement distributions, poses unique challenges for those who wish to resolve these sex differences. First of all, the previously held assumption that countries’ positive equality policies are *particularly* good for girls’ mathematics achievement is inconsistent with the finding that both sexes have higher mathematics achievement in countries with these policies (Table 3). One possibility is that the relation between equality policies and achievement is due to overall living standards and not these policies per se [16] (note that in cross-national studies, it can be difficult to distinguish between outcomes of improved equality measures and outcomes of economic development, because these two are often related). Girls’ achievement clearly improves as living standards improve, but the gains are slightly higher for boys: Across- and within-nations, not only is the sex difference in reading smaller at the high end in countries with higher living standards, high-achieving boys have higher scores than high-achieving girls in mathematics. Researchers do not yet agree on why this pattern occurs. One possibility is that the greater variation in boys’ than girls’ cognitive ability [21]–[22] combined with a sex difference in sensitivity to living conditions contributes to the pattern of performance across the continuum [10](pp.313,411–412) [4]. One predicted result is that improvements in living conditions will benefit boys’ achievement across the continuum more than girls’ achievement, whereas deteriorating conditions will adversely affect boys more than girls.

The finding that countries with higher living standards showed larger sex differences in mathematics is similar to that found for spatial cognition and matches the conclusion of Fryer and Levitt’s study [17] of the sex difference in mathematics in U.S. children mentioned earlier. Lippa and colleagues [23] found that sex differences on tasks measuring spatial abilities were larger in higher-developed countries. Halpern [11](p.337–340) suggests that this finding might be an example of the “Matthew effect” [24]: When there are small differences between boys and girls at a young age, these differences will grow the more resources are targeted at improving children’s skills in the areas in which there is a difference. Nevertheless, a simple “Matthew effect” itself does not explain and seems inconsistent with the finding that the sex difference in mathematics is larger at the high end of the performance continuum while the sex difference in reading is smaller at the high end of the performance continuum (one would expect that both these effects would be larger at the high end). Whatever the contributing factors to international variation in sex differences in mathematics achievement, the implication is that reducing the sex difference in mathematics achievement is not simply a matter of national policy focused on improving girls’ achievement.

What do these findings mean for policy makers or educators who wish to reduce the sex differences in mathematics performance, in particular the underrepresentation of women in fields such as mathematics? Given our finding that nations’ rankings on equality policies are not consistently predictive of sex differences in mathematics achievement, educators and policy makers might reconsider the extent to which the current underrepresentation is related to equality issues. In regard to this point, Ceci and Williams [25] noted that the nature of issues keeping women out of STEM has changed over the years. While there are now more women among the highest performers in mathematics among young adults than 3 decades ago, the sex ratio among the highest performers has been stable for the last 2 decades [26], further questioning whether a change in socio-cultural factors will affect this ratio (as suggested elsewhere [13] [27]). Therefore, we think that it is important to address issues that are independent of equality measures. For example, the strong gender difference in vocational interests between men and women is not an equality issue, but is a matter of individual differences that have been stable for many decades [28]–[30]. Potentially, focusing on differences in how boys and girls learn and how they become interested in STEM topics is important to consider when developing interventions; policy makers could also benefit from working closer together with career counseling psychologists who have developed theoretical and methodological frameworks to take individual differences into account [31]–[32]. In sum, we conclude that we urgently need more research in exactly which factors contribute to sex differences in scholastic achievement and career choice.

The implications for policy makers or educators who wish to reduce the sex differences in reading performance are different. Whereas sex differences in mathematics may be related to the male advantage in spatial abilities [4], girls advantage in reading (and writing) may be related to an early advantage in many language-related competencies that facilitate learning to read (for a review see pp.119–122 of [11]). Further, reading comprehension might also require more complex underlying social-cognitive processes for which girls also have an advantage [10](p.413), such as perspective taking, “theory of mind”, and social understanding [33]–[34]. Given that the sex differences in reading-related skills are evident from a young age, it is likely to have an accumulative negative effect on boys’ reading development, and in that context, intervention should focus on young boys.

Further, it is important to distinguish between the benefits of national prosperity on scholastic achievement of girls on the one hand and the sex differences in scholastic achievement on the other hand. These are two separate issues that are easy to conflate. Although it is true that women’s achievement is higher in economically developed countries than in less economically developed countries (Table S4), it is *not sufficient* to point to the benefits of advanced development for women’s achievement, as some authors have done. This because it benefits both sexes, and thus, conflating the advantages of strong economic development with reducing the continuing sex difference in mathematics performance might be counter-productive. In fact, within countries, it is the high-achieving female students who score lower than high-achieving male students; this illustrates that overall achievement and the sex differences in performance are two entirely different topics. Altogether, there is no reason to believe that improving living standards and overall achievement will reduce the sex difference in mathematics performance.

Related to this latter problem is that the increased participation of women in higher education might actually obscure the continuing underrepresentation of women choosing a career in STEM. This is because the number of women attending college has increased much faster than that of men; for example, in the U.S. the percentage of women enrolling in college increased from 42% in 1970 to 56% in 2000 [35]; numbers in the U.K. are similar [36]. The faster growth in the female than in the male student population has, obviously, resulted in gender-ratios closer to 50% within a number of fields. Importantly, however, this does not mean that women’s interest in mathematics has changed compared to men’s interest in mathematics. In order to determine whether this is the case, an analysis of the fraction of women majoring in mathematics as a fraction of all enrolling women (in all subjects) is necessary, a statistic that is rarely reported in studies of gender distributions. Our own analysis (Figure S1) of the fraction of women that enrolled in a mathematics degree program at universities in the United Kingdom and The Netherlands (both countries have these numbers publicly available) shows that the relation between the fraction of female mathematics students as a proportion of all female students and the fraction of male mathematics students as a proportion of all male students has remained constant over the last 20 years in the Netherlands and the last 10 years in the United Kingdom. Therefore, we conclude that while equality may increase female participation in higher education, it has no noticeable effect on the underrepresentation of women in mathematics. This might help to explain why we still have few women reaching the top in this field (of course, our analysis of this is not the main point of this article, but it is an important topic for further study).

In summary, there are two distinct sex differences in scholastic performance that affect very different segments of the population. On the one hand, boys score lower in reading, in particular at the low end of the reading performance continuum. On the other hand, girls score lower in mathematics at the high end of the mathematics performance continuum. It is important to realize that the latter phenomenon continues to exist, despite the educational gains of women in economically developed countries, and the increased participation of women in higher education in general can easily give the false impression that we are getting closer to the end of the sex difference in mathematics. Our data show that it is important to consider the two types of sex differences separately. On the one hand, if policy makers and educators wish to reduce these sex differences in performance, they need to focus on the higher-achieving girls, and they need to look beyond traditional equality issues and invest in research in how other factors, such as interest differences contribute to the sex differences in performance. Further, the relatively ignored situation for reading and boys seems entirely different. Sex differences in reading are not only persistent and growing, they are particularly large for the most vulnerable boys at the bottom of the reading performance continuum. Addressing this situation will likely require a very different approach than that needed to reduce sex differences in mathematics performance.

## Materials and Methods

### PISA Material

The Programme for International Student Assessment (PISA) conducted four separate assessments (in 2000, 2003, 2006, and 2009). All PISA data, guidance for data analysis, and reports are freely available from http://www.pisa.oecd.org (accessed 2013 Feb 2). Here, we summarize the most essential aspects of the data.

The number of countries contributing to the PISA data sets include both OECD and OECD-partner countries. The number of participating countries/regions (e.g., Hong Kong) has increased to 74 in 2009 (Table S5). The aim of PISA is to measure reading, mathematical, and scientific skills in 15 year olds (the exact age for inclusion is 15 years and 3 months to 16 years and 2 months). The test (which takes an individual student 2 hours) aims to capture how well students can apply their knowledge in the domains of reading, mathematics, and science, and not to merely test what students have learned in their specific national and school curriculum.

*Mathematics* questions are often set in applied settings, for example “A pizzeria serves two round pizzas of the same thickness in different sizes. The smaller one has a diameter of 30 cm and costs 30 zeds. The larger one has a diameter of 40 cm and costs 40 zeds. Which pizza is better value for money? Show your reasoning.” or “Nick wants to pave the rectangular patio of his new house. The patio has length 5.25 metres and width 3.00 metres. He needs 81 bricks per square metre. Calculate how many bricks Nick needs for the whole patio.” *Reading* questions typically provide a short text (which can be as short as a few sentences) followed by a question that requires an understanding about the intent of the writer, relationships between the concepts in the text, etc. These and other sample questions can be viewed via http://pisa-sq.acer.edu.au (accessed 2013 Feb 2).

PISA selects a representative sample of schools and students from each participating country. Each student’s scores in the different domains (mathematics, reading, science) are scaled such that the average of students in OECD countries is 500 points and the standard deviation is 100 points. The exact details of how average country scores are calculated is not relevant for understanding the current analyses. We would like to point out that PISA gives detailed guidance on how to perform data analyses [37], and we have strictly followed these guidelines. In short, PISA provides multiple scores (5 different *plausible values* for reading and mathematics) and weights for each student, such that a representative score of each nation and gender can be calculated [37]. The plausible values (this is a statistical concept [38]) reflect the fact that PISA uses item response theory to estimate student’s performance. The use of these values helps to take into consideration that different students perform different test items due to the rotating test design underlying PISA assessments (i.e., not all students perform exactly the same test items, which helps to increase the range of test items and to keep the test duration within practicable limits).

Sex differences in mathematics performance are calculated by subtracting the boys’ and girls’ scores (Table S1). Higher values mean a larger disadvantage of girls. Sex differences in reading performance are calculated by subtracting the girls’ and boys’ scores; here, higher values mean a larger disadvantage of boys (note that there is not a single country where boys had a higher reading score than girls, Table S1).

In order to calculate whether or not a sex difference within a country is statistically significant (*p*<.05), we calculated the standard errors of the difference in accordance with the prescribed procedure of the PISA manual [37, p.137].

We downloaded the human development and equality indicators (Table S6) from the Human Development Report Office (HDRO) of the United Nations (http://hdr.undp.org/, accessed 2013 Feb 2) and the World Economic Forum (http://www.weforum.org/issues/global-gender-gap/, accessed 2013 Feb 2). These include: 1) The Human Development Index (HDI), which reflects the living standard, and is based on people’s health, knowledge, and income. 2) The Gender Inequality Index (GII), which reflects inequality between the two sexes and takes mortality, fertility, representation in parliament, education, and the equal representation in the labor market into account. 3) The Gender Development Index (GDI), which is similar to the HDI, but correcting for gender inequalities. 4) The Gender Empowerment Measure (GEM), which takes women’s role in politics and the economy into account. 5) The Global Gender Gap Index (GGGI), which reflects sex differences in participation in the economy (e.g., income), education, health, and the gender ratio of politicians at various levels in the political hierarchy. 6) The Gini coefficient, which reflects general equality in society (with higher values reflecting less equal societies).

Most of these variables are not normally distributed; only the GEM and GGGI are not significantly different from the normal distribution (as tested with the Shapiro Wilks test of normality). For correlational analyses (Tables 3, S4), we used the Pearson coefficient for normally distributed variables and Spearman rank correlation for the others. We choose the data of 2008 (because not all years are available).

### Data Analysis

We used the PSPP (http://www.gnu.org/software/pspp/, accessed 2013 Feb 2) software package to load the raw data files using the SPSS scripts provided by PISA. We then saved them in data files that can be read into the statistical software package R (http://www.r-project.org, accessed 2013 Feb 2); all further analyses were carried out in R using the Advanced Research Computing facilities at the University of Leeds.

The curves in Figure 3 are calculated as follows. The curves are based on the countries that participated in all four PISA assessments (2000, 2003, 2006, and 2009). First, we calculated for each assessment and each country the performance percentiles for boys and girls separately and then averaged across the 33 nations that participated in all 4 assessments. Next, for each assessment, we calculated the sex differences in the performance percentiles by subtracting the boys’ and girls’ performance percentiles similar to other calculations of sex differences. That is, for the sex differences in mathematics we subtract the girls’ from the boys’ scores and for the sex differences in reading we subtract the boys’ scores from the girls’ scores.

## Supporting Information

Figure S1 **Enrollment in mathematics at Dutch and British universities.** The percentage of Dutch male first year students enrolled in a mathematics degree program as a proportion of all first-year male students enrolling in all subjects (blue) at university. Same for Dutch female students (red). Same for UK students (+ symbol). Note that the relative proportion of female compared to male students stayed relatively constant, suggesting that the interest of female students compared to male students stayed similar. Data from the British *Higher Education Information Database (HEIDI* http://www.hesa.ac.uk *)* and the Dutch Statistics Netherlands (http://www.cbs.nl). In 2011, within mathematics, the ratio of first year male to female mathematics students is currently 2.23∶1 in The Netherlands, and 1.63∶1 in the U.K.
(TIFF)

Table S1 Total scores and sex differences in mathematics and reading by country and assessment year. For each country and each assessment, the average scores of boys and girls are listed (Total). For sex differences (abbreviated as “Diff”) in mathematics, a negative number indicates girls outperformed boys. For sex differences in reading, all numbers are positive (i.e., girls always outperformed boys). If a difference is in *bold italic font*, it is statistically significant (*p*<.05).
(DOC)

Table S2 Sex difference in mathematics in all participating countries. The first set of scores compares boys and girls at the same points on the gender-specific achievement distributions. Comparing the bottom 5% of boys (relative to all other boys) to the bottom 5% of girls (relative to all other girls), the difference in mathematics achievement ranges from a 1.1 point advantage for girls (2003) to a 1.1 point advantage for boys (2006). The second set of scores is the ratio of boys to girls at various percentiles of overall (including both genders) achievement.
(DOC)

Table S3 Sex difference in reading in all participating countries. The first set of scores compares boys and girls at the same points on the gender-specific achievement distributions. Comparing the bottom 5% of boys (relative to all other boys) to the bottom 5% of girls (relative to all other girls), the advantage of girls ranges from 40.8 points (2000) to 50.3 points (2009). The second set of scores is the ratio of boys to girls at various percentiles of overall (including both genders) achievement.
(DOC)

Table S4 Correlations between mathematics (top) and reading scores (bottom) and human development and equality indicators. HDI: Human Development Index. GII = Gender Inequality Index. GDI = Gender Development Index. GEM = Gender Empowerment Measure. GGGI = Global Gender sex difference Index. Gini = Gini coeffient. Stars indicate significance level: *p<.05; ** *p*<.01; ***p<.001.
(DOC)

Table S5 Sample sizes for participating countries and economic regions.
(DOC)

Table S6 Human development and gender equality scores. HDI: Human Development Index. GII = Gender Inequality Index. GDI = Gender Development Index. GEM = Gender Empowerment Measure. GGGI = Global Gender Gap Index. Gini = Gini coeffient. We correlated the magnitude of the sex differences in mathematics and reading for each year with each of these variables to determine if a consistent pattern of correlation emerged (i.e., the sex differences in mathematics in each PISA assessment correlates with the variable). No such pattern was found (Table 3).
(DOC)
