# Supplementary figures and images for: Sex Differences in Mathematics and Reading Achievement Are Inversely Related: Within- and Across-Nation Assessment of 10 Years of PISA Data
Source: PLoS One. 2013 Mar 13;8(3):e57988. doi: 10.1371/journal.pone.0057988 (PMC3596327; doi:10.1371/journal.pone.0057988)

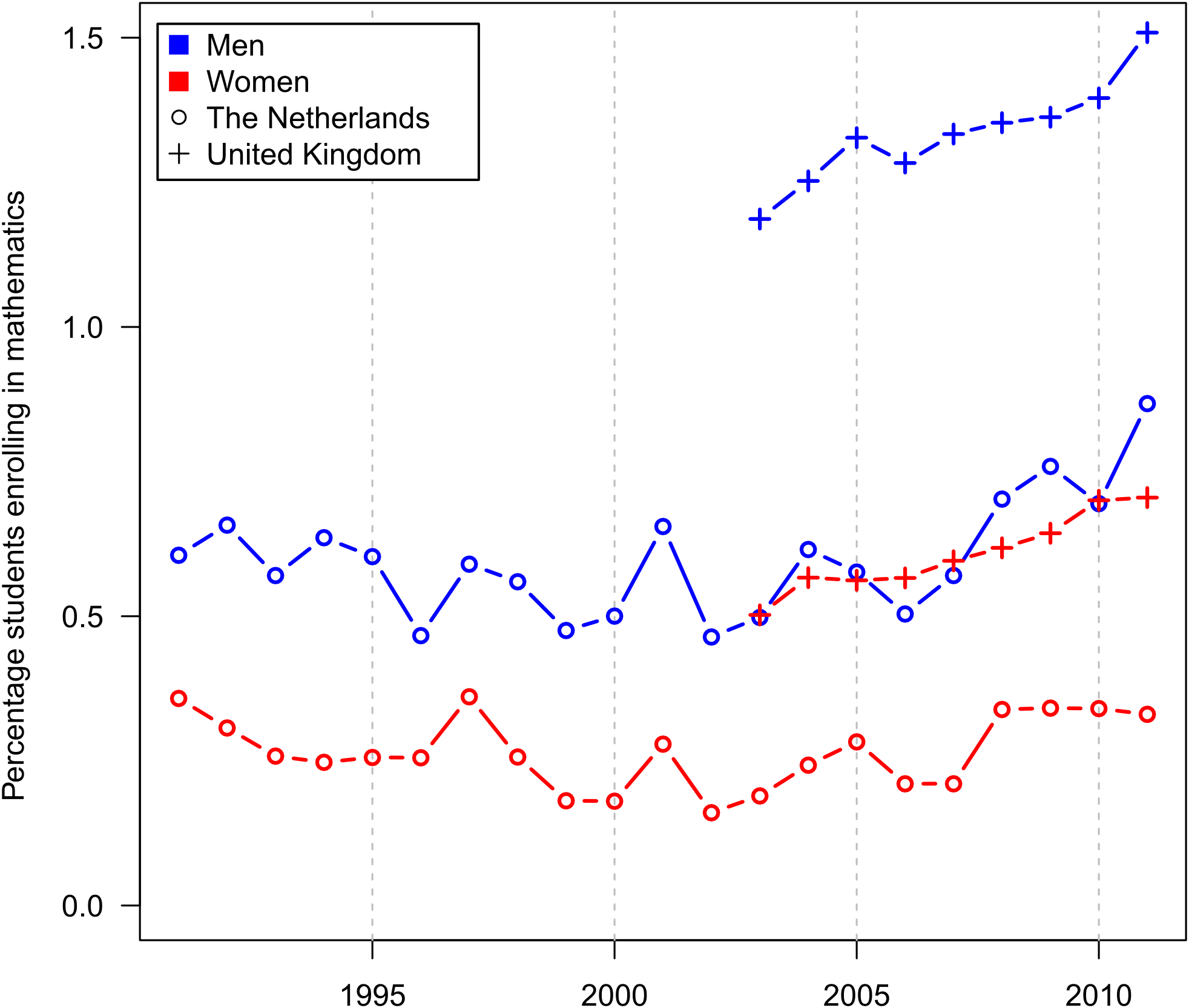

Supplement: Figure S1 — Enrollment in mathematics at Dutch and British universities. The percentage of Dutch male first year students enrolled in a mathematics degree program as a proportion of all first-year male students enrolling in all subjects (blue) at university. Same for Dutch female students (red). Same for UK students (+ symbol). Note that the relative proportion of female compared to male students stayed relatively constant, suggesting that the interest of female students compared to male students stayed similar. Data from the British Higher Education Information Database (HEIDI http://www.hesa.ac.uk ) and the Dutch Statistics Netherlands (http://www.cbs.nl). In 2011, within mathematics, the ratio of first year male to female mathematics students is currently 2.23∶1 in The Netherlands, and 1.63∶1 in the U.K. (TIFF) [file pone.0057988.s001.tif]
