# Supplementary material for: Sex Differences in Mathematics and Reading Achievement Are Inversely Related: Within- and Across-Nation Assessment of 10 Years of PISA Data
Source: PLoS One. 2013 Mar 13;8(3):e57988. doi: 10.1371/journal.pone.0057988 (PMC3596327; doi:10.1371/journal.pone.0057988)
Supplement: Table S1 — Total scores and sex differences in mathematics and reading by country and assessment year. For each country and each assessment, the average scores of boys and girls are listed (Total). For sex differences (abbreviated as “Diff”) in mathematics, a negative number indicates girls outperformed boys. For sex differences in reading, all numbers are positive (i.e., girls always outperformed boys). If a difference is in bold italic font, it is statistically significant (p<.05). (DOC) [file pone.0057988.s002.doc]

| **Country** | **Mathematics** | | | | | | | | **Reading** | | | | | | | |
| --- | --- | --- | --- | --- | --- | --- | --- | --- | --- | --- | --- | --- | --- | --- | --- | --- |
|  | 2000 | | 2003 | | 2006 | | 2009 | | 2000 | | 2003 | | 2006 | | 2009 | |
|  | Total | Diff | Total | Diff | Total | Diff | Total | Diff | Total | Diff | Total | Diff | Total | Diff | Total | Diff |
| Australia | 533 | 12 | 524 | 5 | 520 | ***14*** | 514 | ***10*** | 530 | ***33*** | 526 | ***39*** | 514 | ***37*** | 514 | ***37*** |
| Austria | 502 | ***20*** | 506 | 7 | 506 | ***23*** | 496 | ***20*** | 492 | ***33*** | 490 | ***47*** | 490 | ***45*** | 470 | ***41*** |
| Belgium | 521 | 6 | 529 | 8 | 520 | 7 | 515 | ***22*** | 508 | ***33*** | 508 | ***37*** | 502 | ***40*** | 506 | ***27*** |
| Canada | 534 | ***10*** | 536 | ***11*** | 527 | ***14*** | 527 | ***12*** | 535 | ***32*** | 530 | ***32*** | 527 | ***32*** | 524 | ***35*** |
| Czech | 498 | ***12*** | 516 | ***15*** | 509 | 10 | 492 | 5 | 492 | ***37*** | 488 | ***31*** | 486 | ***46*** | 480 | ***48*** |
| Denmark | 514 | ***15*** | 514 | ***17*** | 513 | ***10*** | 503 | ***16*** | 498 | ***25*** | 492 | ***26*** | 494 | ***29*** | 494 | ***29*** |
| Finland | 536 | 1 | 544 | ***7*** | 548 | ***11*** | 540 | 3 | 546 | ***51*** | 543 | ***44*** | 546 | ***51*** | 536 | ***55*** |
| France | 518 | ***14*** | 511 | ***8*** | 496 | 7 | 497 | ***16*** | 504 | ***29*** | 495 | ***38*** | 488 | ***35*** | 495 | ***40*** |
| Germany | 490 | ***15*** | 504 | ***9*** | 504 | ***19*** | 512 | ***15*** | 485 | ***34*** | 492 | ***42*** | 496 | ***42*** | 498 | ***40*** |
| Greece | 448 | 7 | 446 | ***19*** | 460 | 5 | 466 | ***14*** | 474 | ***37*** | 472 | ***37*** | 460 | ***56*** | 482 | ***47*** |
| Hungary | 488 | 7 | 490 | ***8*** | 491 | ***10*** | 490 | ***12*** | 480 | ***31*** | 482 | ***31*** | 483 | ***40*** | 494 | ***38*** |
| Iceland | 516 | -5 | 516 | ***-15*** | 506 | -5 | 506 | 3 | 508 | ***40*** | 493 | ***58*** | 484 | ***49*** | 500 | ***44*** |
| Ireland | 504 | ***13*** | 502 | ***15*** | 502 | ***11*** | 487 | 8 | 528 | ***29*** | 516 | ***29*** | 517 | ***34*** | 496 | ***39*** |
| Italy | 458 | 8 | 466 | ***18*** | 462 | ***17*** | 482 | ***15*** | 488 | ***38*** | 475 | ***40*** | 468 | ***41*** | 487 | ***46*** |
| Japan | 557 | 8 | 534 | 9 | 523 | ***20*** | 529 | 10 | 522 | ***30*** | 498 | ***22*** | 498 | ***30*** | 520 | ***39*** |
| Korea | 546 | ***27*** | 540 | ***24*** | 548 | 9 | 546 | 4 | 526 | ***14*** | 536 | ***22*** | 556 | ***35*** | 540 | ***35*** |
| Luxembourg | 446 | ***15*** | 494 | ***17*** | 490 | ***16*** | 489 | ***20*** | 442 | ***27*** | 480 | ***33*** | 480 | ***31*** | 472 | ***39*** |
| Mexico | 388 | ***11*** | 386 | ***11*** | 406 | ***9*** | 418 | ***13*** | 422 | ***21*** | 400 | ***21*** | 410 | ***34*** | 426 | ***25*** |
| New Zealand | 538 | -3 | 524 | ***15*** | 522 | ***10*** | 519 | 8 | 530 | ***46*** | 522 | ***27*** | 520 | ***37*** | 522 | ***45*** |
| Norway | 500 | ***11*** | 495 | 6 | 490 | ***6*** | 498 | 5 | 508 | ***43*** | 500 | ***50*** | 485 | ***46*** | 504 | ***47*** |
| Poland | 470 | 4 | 490 | 6 | 496 | ***9*** | 495 | 4 | 479 | ***36*** | 496 | ***39*** | 508 | ***41*** | 500 | ***49*** |
| Portugal | 455 | ***18*** | 466 | ***12*** | 466 | ***15*** | 487 | ***12*** | 470 | ***24*** | 477 | ***36*** | 472 | ***33*** | 489 | ***38*** |
| Spain | 478 | ***18*** | 486 | ***9*** | 480 | ***8*** | 484 | ***19*** | 493 | ***24*** | 480 | ***39*** | 461 | ***36*** | 482 | ***29*** |
| Sweden | 510 | 7 | 509 | ***6*** | 502 | 5 | 494 | -2 | 518 | ***37*** | 514 | ***37*** | 508 | ***40*** | 498 | ***46*** |
| Switzerland | 530 | ***14*** | 526 | ***17*** | 530 | ***13*** | 534 | ***20*** | 495 | ***30*** | 500 | ***35*** | 500 | ***31*** | 500 | ***39*** |
| United Kingdom | 530 | 8 | 508 | 7 | 496 | ***17*** | 492 | ***21*** | 524 | ***25*** | 506 | ***28*** | 495 | ***30*** | 494 | ***26*** |
| United States | 494 | 7 | 483 | ***6*** | 474 | ***9*** | 487 | ***20*** | 504 | ***28*** | 495 | ***32*** | - | - | 500 | ***25*** |
| Albania | 381 | ***-18*** | - | - | - | - | 378 | ***-11*** | 348 | ***59*** | - | - | - | - | 386 | ***62*** |
| Argentina | 388 | -3 | - | - | 382 | ***13*** | 388 | ***11*** | 415 | ***44*** | - | - | 372 | ***54*** | 397 | ***36*** |
| Brazil | 336 | ***27*** | 356 | ***17*** | 370 | ***19*** | 386 | ***15*** | 396 | ***16*** | 402 | ***35*** | 392 | ***32*** | 411 | ***28*** |
| Bulgaria | 430 | -4 | - | - | 414 | -3 | 428 | -4 | 431 | ***48*** | - | - | 403 | ***58*** | 430 | ***61*** |
| Chile | 384 | 8 | - | - | 410 | ***28*** | 420 | ***21*** | 408 | ***25*** | - | - | 442 | ***17*** | 450 | ***22*** |
| Hong Kong | 560 | ***18*** | 550 | 4 | 548 | ***15*** | 554 | ***14*** | 526 | ***15*** | 510 | ***31*** | 536 | ***31*** | 534 | ***32*** |
| Indonesia | 366 | 5 | 360 | 4 | 390 | ***17*** | 372 | -1 | 370 | ***20*** | 382 | ***25*** | 393 | ***18*** | 402 | ***37*** |
| Israel | 436 | 12 | - | - | 442 | 12 | 447 | 8 | 452 | 15 | - | - | 438 | ***43*** | 474 | ***43*** |
| Latvia | 464 | 7 | 484 | 3 | 486 | 5 | 482 | 2 | 458 | ***53*** | 490 | ***39*** | 479 | ***50*** | 484 | ***47*** |
| Liechten-  stein | 516 | 11 | 536 | ***29*** | 525 | 0 | 535 | ***24*** | 484 | ***32*** | 526 | 17 | 508 | ***45*** | 500 | ***32*** |
| Macedonia | 382 | -3 | - | - | - | - | - | - | 374 | ***51*** | - | - | - | - | - | - |
| Peru | 293 | 16 | - | - | - | - | 365 | ***18*** | 327 | 6 | - | - | - | - | 370 | ***22*** |
| Romania | 425 | -10 | - | - | 415 | ***6*** | 427 | 4 | 428 | ***13*** | - | - | 396 | ***44*** | 424 | ***42*** |
| Russia | 478 | -1 | 468 | ***10*** | 476 | 6 | 468 | 2 | 462 | ***38*** | 442 | ***28*** | 439 | ***38*** | 460 | ***45*** |
| Thailand | 432 | -6 | 417 | -4 | 416 | -7 | 419 | 4 | 427 | ***42*** | 418 | ***43*** | 413 | ***54*** | 419 | ***38*** |
| The Netherlands | - | - | 538 | 5 | 530 | ***13*** | 526 | ***17*** | - | - | 514 | ***21*** | 507 | ***24*** | 508 | ***25*** |
| Slovakia | - | - | 498 | ***18*** | 492 | ***14*** | 496 | 3 | - | - | 470 | ***33*** | 467 | ***42*** | 478 | ***51*** |
| Turkey | - | - | 422 | ***15*** | 424 | 6 | 446 | ***11*** | - | - | 442 | ***33*** | 449 | ***44*** | 464 | ***43*** |
| Macao | - | - | 528 | ***21*** | 525 | ***10*** | 526 | ***11*** | - | - | 498 | ***13*** | 492 | ***26*** | 487 | ***34*** |
| Serbia | - | - | 436 | 1 | 436 | 5 | 442 | ***11*** | - | - | 412 | ***43*** | 402 | ***41*** | 442 | ***40*** |
| Tunisia | - | - | 359 | ***12*** | 366 | ***15*** | 372 | ***12*** | - | - | 374 | ***25*** | 380 | ***37*** | 402 | ***31*** |
| Uruguay | - | - | 422 | ***12*** | 426 | ***13*** | 427 | ***12*** | - | - | 434 | ***39*** | 412 | ***46*** | 424 | ***41*** |
| Azerbaijan | - | - | - | - | 476 | -2 | 431 | ***8*** | - | - | - | - | 353 | ***20*** | 362 | ***24*** |
| Chinese Taipei | - | - | - | - | 550 | ***13*** | 544 | 5 | - | - | - | - | 496 | ***21*** | 496 | ***37*** |
| Colombia | - | - | - | - | 371 | ***22*** | 382 | ***32*** | - | - | - | - | 384 | ***19*** | 413 | ***10*** |
| Croatia | - | - | - | - | 468 | ***13*** | 460 | ***11*** | - | - | - | - | 477 | ***50*** | 478 | ***51*** |
| Estonia | - | - | - | - | 514 | 1 | 512 | ***8*** | - | - | - | - | 501 | ***46*** | 502 | ***44*** |
| Jordan | - | - | - | - | 384 | -7 | 386 | -1 | - | - | - | - | 400 | ***55*** | 406 | ***57*** |
| Kyrgyzstan | - | - | - | - | 310 | 1 | 331 | ***-6*** | - | - | - | - | 282 | ***51*** | 314 | ***53*** |
| Lithuania | - | - | - | - | 486 | 2 | 477 | ***-6*** | - | - | - | - | 470 | ***51*** | 468 | ***59*** |
| Montenegro | - | - | - | - | 399 | ***12*** | 402 | ***12*** | - | - | - | - | 392 | ***45*** | 408 | ***52*** |
| Qatar | - | - | - | - | 318 | ***-14*** | 368 | ***-5*** | - | - | - | - | 313 | ***66*** | 372 | ***50*** |
| Slovenia | - | - | - | - | 504 | 5 | 502 | 1 | - | - | - | - | 494 | ***54*** | 484 | ***55*** |
| Georgia | - | - | - | - | - | - | 380 | -3 | - | - | - | - | - | - | 374 | ***61*** |
| Costa Rica | - | - | - | - | - | - | 410 | ***26*** | - | - | - | - | - | - | 442 | ***14*** |
| Himachal Pradesh (I) | - | - | - | - | - | - | 339 | ***30*** | - | - | - | - | - | - | 317 | ***10*** |
| Kazakhstan | - | - | - | - | - | - | 405 | 0 | - | - | - | - | - | - | 390 | ***43*** |
| Malaysia | - | - | - | - | - | - | 404 | -3 | - | - | - | - | - | - | 414 | ***35*** |
| Malta | - | - | - | - | - | - | 462 | ***-15*** | - | - | - | - | - | - | 442 | ***72*** |
| Mauritius | - | - | - | - | - | - | 420 | 4 | - | - | - | - | - | - | 406 | ***40*** |
| Miranda (Venezuela) | - | - | - | - | - | - | 398 | ***17*** | - | - | - | - | - | - | 421 | ***18*** |
| Moldova | - | - | - | - | - | - | 398 | 3 | - | - | - | - | - | - | 388 | ***45*** |
| Panama | - | - | - | - | - | - | 360 | 5 | - | - | - | - | - | - | 370 | ***33*** |
| Shanghai-China | - | - | - | - | - | - | 600 | -2 | - | - | - | - | - | - | 556 | ***40*** |
| Singapore | - | - | - | - | - | - | 562 | ***6*** | - | - | - | - | - | - | 526 | ***31*** |
| Tamil Nadu (India) | - | - | - | - | - | - | 350 | -7 | - | - | - | - | - | - | 335 | ***36*** |
| Trinidad and Tobago | - | - | - | - | - | - | 414 | ***-8*** | - | - | - | - | - | - | 416 | ***58*** |
| United Arab Emirates | - | - | - | - | - | - | 421 | ***-6*** | - | - | - | - | - | - | 431 | ***58*** |
