# Supplementary material for: Sex Differences in Mathematics and Reading Achievement Are Inversely Related: Within- and Across-Nation Assessment of 10 Years of PISA Data
Source: PLoS One. 2013 Mar 13;8(3):e57988. doi: 10.1371/journal.pone.0057988 (PMC3596327; doi:10.1371/journal.pone.0057988)
Supplement: Table S2 — Sex difference in mathematics in all participating countries. The first set of scores compares boys and girls at the same points on the gender-specific achievement distributions. Comparing the bottom 5% of boys (relative to all other boys) to the bottom 5% of girls (relative to all other girls), the difference in mathematics achievement ranges from a 1.1 point advantage for girls (2003) to a 1.1 point advantage for boys (2006). The second set of scores is the ratio of boys to girls at various percentiles of overall (including both genders) achievement. (DOC) [file pone.0057988.s003.doc]

| **Achievement Percentile** | **2000** | **2003** | **2006** | **2009** |
| --- | --- | --- | --- | --- |
| **Sex difference in mathematics** |  |  |  |  |
| **(Boys’ Scores – Girls’ Scores)** |  |  |  |  |
| 5^th^ | 0.5 | -1.1 | 1.1 | 0.1 |
| 50^th^ | 8.5 | 10.6 | 8.9 | 8.1 |
| 95^th^ | 17.4 | 22.2 | 18.9 | 17.0 |
| Mean | 8.1 | 10.3 | 9.3 | 8.3 |
| **Ratio of Boys to Girls** |  |  |  |  |
| 1^st^ | 1.1 | 1.2 | 1.3 | 1.2 |
| 5^th^ | 1.0 | 1.1 | 1.0 | 1.0 |
| 95^th^ | 1.7 | 1.9 | 1.6 | 1.6 |
| 99^th^ | 2.5 | 2.6 | 2.3 | 2.1 |

Note: For the mathematics sex difference, Liechtenstein did not have any girls at the <1st (2003) or >99th percentiles (2003, 2009). For the boys/girls ratios for the 1st percentile, there are no girls for Liechtenstein in 2000, 2003, 2009; Macedonia (2000) and Turkey (2003) also had no girls in this end of the distribution.
