# Supplementary material for: Sex Differences in Mathematics and Reading Achievement Are Inversely Related: Within- and Across-Nation Assessment of 10 Years of PISA Data
Source: PLoS One. 2013 Mar 13;8(3):e57988. doi: 10.1371/journal.pone.0057988 (PMC3596327; doi:10.1371/journal.pone.0057988)
Supplement: Table S3 — Sex difference in reading in all participating countries. The first set of scores compares boys and girls at the same points on the gender-specific achievement distributions. Comparing the bottom 5% of boys (relative to all other boys) to the bottom 5% of girls (relative to all other girls), the advantage of girls ranges from 40.8 points (2000) to 50.3 points (2009). The second set of scores is the ratio of boys to girls at various percentiles of overall (including both genders) achievement. (DOC) [file pone.0057988.s004.doc]

| **Achievement Percentile** | **2000** | **2003** | **2006** | **2009** |
| --- | --- | --- | --- | --- |
| **Sex difference in Reading** |  |  |  |  |
| **(Girls’ Scores – Boys’ Scores)** |  |  |  |  |
| 5^th^ | 40.8 | 48.5 | 52.9 | 50.3 |
| 50^th^ | 31.9 | 32.8 | 39.8 | 40.4 |
| 95^th^ | 20.0 | 21.3 | 25.6 | 27.1 |
| Mean | 32.0 | 33.6 | 39.7 | 39.8 |
| **Ratio of Boys to Girls** |  |  |  |  |
| 1^st^ | 3.0 | 4.0 | 6.9 | 6.4 |
| 5^th^ | 2.5 | 2.8 | 3.1 | 3.4 |
| 95^th^ | 0.6 | 0.6 | 0.5 | 0.5 |
| 99^th^ | 0.5 | 0.6 | 0.5 | 0.5 |
