# Supplementary material for: Sex Differences in Mathematics and Reading Achievement Are Inversely Related: Within- and Across-Nation Assessment of 10 Years of PISA Data
Source: PLoS One. 2013 Mar 13;8(3):e57988. doi: 10.1371/journal.pone.0057988 (PMC3596327; doi:10.1371/journal.pone.0057988)
Supplement: Table S4 — Correlations between mathematics (top) and reading scores (bottom) and human development and equality indicators. HDI: Human Development Index. GII = Gender Inequality Index. GDI = Gender Development Index. GEM = Gender Empowerment Measure. GGGI = Global Gender sex difference Index. Gini = Gini coeffient. Stars indicate significance level: *p<.05; ** p<.01; ***p<.001. (DOC) [file pone.0057988.s005.doc]

|  | **Mathematics performance** | | | | | | | |
| --- | --- | --- | --- | --- | --- | --- | --- | --- |
|  | 2000 | | 2003 | | 2006 | | 2009 | |
|  | Girls | Boys | Girls | Boys | Girls | Boys | Girls | Boys |
| HDI | 0.77 *** | 0.81 *** | 0.82 *** | 0.83 *** | 0.79 *** | 0.81 *** | 0.75 *** | 0.77 *** |
| GII | -0.80 *** | -0.82 *** | -0.85 *** | -0.86 *** | -0.85 *** | -0.85 *** | -0.86 *** | -0.85 *** |
| GDI | 0.79 *** | 0.82 *** | 0.83 *** | 0.84 *** | 0.80 *** | 0.83 *** | 0.75 *** | 0.77 *** |
| GEM | 0.59 *** | 0.60 *** | 0.69 *** | 0.66 *** | 0.60 *** | 0.61 *** | 0.54 *** | 0.57 *** |
| GGGI | 0.44 ** | 0.42 ** | 0.55 *** | 0.49 ** | 0.55 *** | 0.54 *** | 0.46 *** | 0.47 *** |
| Gini | -0.62 *** | -0.59 *** | -0.60 *** | -0.60 *** | -0.58 *** | -0.54 *** | -0.54 *** | -0.48 *** |
|  | **Reading performance** | | | | | | | |
|  | 2000 | | 2003 | | 2006 | | 2009 | |
|  | Girls | Boys | Girls | Boys | Girls | Boys | Girls | Boys |
| HDI | 0.85 *** | 0.85 *** | 0.87 *** | 0.85 *** | 0.84 *** | 0.84 *** | 0.82 *** | 0.81 *** |
| GII | -0.81 *** | -0.78 *** | -0.84 *** | -0.81 *** | -0.81 *** | -0.78 *** | -0.82 *** | -0.78 *** |
| GDI | 0.87 *** | 0.86 *** | 0.88 *** | 0.86 *** | 0.84 *** | 0.84 *** | 0.82 *** | 0.81 *** |
| GEM | 0.67 *** | 0.64 *** | 0.72 *** | 0.67 *** | 0.61 *** | 0.59 *** | 0.58 *** | 0.59 *** |
| GGGI | 0.52 *** | 0.45 ** | 0.60 *** | 0.52 *** | 0.51 *** | 0.50 *** | 0.48 *** | 0.48 *** |
| Gini | -0.53 *** | -0.47 ** | -0.50 ** | -0.49 ** | -0.45 ** | -0.40 ** | -0.47 *** | -0.35 ** |
