# Supplementary material for: Sex Differences in Mathematics and Reading Achievement Are Inversely Related: Within- and Across-Nation Assessment of 10 Years of PISA Data
Source: PLoS One. 2013 Mar 13;8(3):e57988. doi: 10.1371/journal.pone.0057988 (PMC3596327; doi:10.1371/journal.pone.0057988)
Supplement: Table S5 — Sample sizes for participating countries and economic regions. (DOC) [file pone.0057988.s006.doc]

| **Country/Region** | **PISA 2000** | | **PISA 2003** | | **PISA 2006** | | **PISA 2009** | |
| --- | --- | --- | --- | --- | --- | --- | --- | --- |
|  | Boys | Girls | Boys | Girls | Boys | Girls | Boys | Girls |
| Australia | 2665 | 2475 | 6335 | 6216 | 7192 | 6978 | 7020 | 7231 |
| Austria | 2344 | 2339 | 2312 | 2285 | 2480 | 2447 | 3252 | 3338 |
| Belgium | 3349 | 3269 | 4597 | 4199 | 4626 | 4231 | 4345 | 4156 |
| Canada | 14726 | 14651 | 13469 | 13748 | 11104 | 11542 | 11431 | 11776 |
| Czech Republic | 2482 | 2866 | 3238 | 3082 | 3146 | 2786 | 3115 | 2949 |
| Denmark | 2113 | 2099 | 2082 | 2136 | 2201 | 2331 | 2886 | 3038 |
| Finland | 2357 | 2507 | 2867 | 2929 | 2329 | 2385 | 2856 | 2954 |
| France | 2290 | 2359 | 2033 | 2267 | 2292 | 2424 | 2087 | 2211 |
| Germany | 2438 | 2574 | 2299 | 2315 | 2491 | 2400 | 2545 | 2434 |
| Greece | 2351 | 2288 | 2261 | 2365 | 2432 | 2441 | 2412 | 2557 |
| Hungary | 2468 | 2385 | 2516 | 2249 | 2286 | 2204 | 2294 | 2311 |
| Iceland | 1650 | 1674 | 1729 | 1621 | 1877 | 1912 | 1792 | 1854 |
| Ireland | 1841 | 1988 | 1973 | 1907 | 2264 | 2321 | 1973 | 1964 |
| Italy | 2413 | 2543 | 5616 | 6023 | 10934 | 10839 | 15696 | 15209 |
| Japan | 2652 | 2604 | 2304 | 2402 | 3003 | 2949 | 3126 | 2962 |
| Korea | 2755 | 2221 | 3211 | 2233 | 2613 | 2563 | 2590 | 2399 |
| Luxembourg | 1737 | 1745 | 1931 | 1992 | 2306 | 2261 | 2319 | 2303 |
| Mexico | 2250 | 2240 | 13814 | 16167 | 14188 | 16783 | 18209 | 20041 |
| New Zealand | 1863 | 1787 | 2286 | 2224 | 2350 | 2473 | 2396 | 2247 |
| Norway | 2068 | 2014 | 2052 | 2012 | 2415 | 2277 | 2375 | 2285 |
| Poland | 1917 | 1737 | 2180 | 2203 | 2719 | 2828 | 2443 | 2474 |
| Portugal | 2156 | 2394 | 2208 | 2400 | 2425 | 2684 | 3020 | 3278 |
| Spain | 2983 | 3096 | 5243 | 5547 | 9803 | 9801 | 13141 | 12746 |
| Sweden | 2230 | 2153 | 2341 | 2283 | 2282 | 2161 | 2311 | 2256 |
| Switzerland | 3034 | 3012 | 4341 | 4079 | 6249 | 5943 | 6020 | 5790 |
| UK | 4641 | 4591 | 4663 | 4872 | 6523 | 6629 | 6062 | 6117 |
| USA | 1824 | 2021 | 2740 | 2715 | 2839 | 2771 | 2687 | 2546 |
| Albania | 2381 | 2577 |  |  |  |  | 2321 | 2275 |
| Argentina | 1868 | 2115 |  |  | 1981 | 2358 | 2183 | 2591 |
| Brazil | 2305 | 2535 | 2059 | 2391 | 4258 | 5037 | 9101 | 11026 |
| Bulgaria | 2439 | 2218 |  |  | 2320 | 2178 | 2231 | 2276 |
| Chile | 2244 | 2640 |  |  | 2830 | 2403 | 2870 | 2799 |
| Hong Kong | 2197 | 2208 | 2219 | 2259 | 2294 | 2351 | 2557 | 2280 |
| Indonesia | 3644 | 3724 | 5309 | 5447 | 5291 | 5356 | 2534 | 2602 |
| Israel | 2054 | 2427 |  |  | 2204 | 2380 | 2648 | 3113 |
| Latvia | 1837 | 2009 | 2229 | 2398 | 2286 | 2433 | 2175 | 2327 |
| Liechtenstein | 156 | 154 | 170 | 162 | 155 | 184 | 181 | 148 |
| Macedonia | 2300 | 2168 |  |  |  |  |  |  |
| Peru | 2161 | 2230 |  |  |  |  | 3000 | 2985 |
| Romania | 2168 | 2661 |  |  | 2684 | 2434 | 2378 | 2398 |
| Russia | 3325 | 3363 | 2887 | 3087 | 2799 | 3000 | 2623 | 2685 |
| Thailand | 2218 | 3122 | 2291 | 2945 | 2608 | 3584 | 2681 | 3544 |
| The Netherlands | 1234 | 1247 | 2015 | 1977 | 2501 | 2370 | 2348 | 2412 |
| Slovakia |  |  | 3732 | 3614 | 2391 | 2340 | 2238 | 2317 |
| Turkey |  |  | 2765 | 2090 | 2652 | 2290 | 2551 | 2445 |
| Macao |  |  | 619 | 631 | 2320 | 2440 | 3011 | 2941 |
| Serbia |  |  | 2215 | 2190 | 2434 | 2364 | 2680 | 2843 |
| Tunisia |  |  | 2325 | 2396 | 2190 | 2450 | 2359 | 2596 |
| Uruguay |  |  | 3082 | 2753 | 2272 | 2567 | 2810 | 3147 |
| Azerbaijan |  |  |  |  | 2685 | 2499 | 2443 | 2248 |
| Chinese Taipei |  |  |  |  | 4620 | 4192 | 2911 | 2920 |
| Colombia |  |  |  |  | 2043 | 2435 | 3711 | 4210 |
| Croatia |  |  |  |  | 2613 | 2600 | 2653 | 2341 |
| Estonia |  |  |  |  | 2479 | 2386 | 2430 | 2297 |
| Jordan |  |  |  |  | 2952 | 3557 | 3120 | 3366 |
| Kyrgyzstan |  |  |  |  | 2731 | 3173 | 2381 | 2605 |
| Lithuania |  |  |  |  | 2384 | 2360 | 2287 | 2241 |
| Montenegro |  |  |  |  | 2330 | 2125 | 2443 | 2382 |
| Qatar |  |  |  |  | 3040 | 3225 | 4510 | 4568 |
| Slovenia |  |  |  |  | 3552 | 3043 | 3333 | 2822 |
| Georgia |  |  |  |  |  |  | 2295 | 2351 |
| Costa Rica |  |  |  |  |  |  | 2142 | 2436 |
| Himachal Pradesh (India) |  |  |  |  |  |  | 804 | 812 |
| Kazakhstan |  |  |  |  |  |  | 2723 | 2689 |
| Malaysia |  |  |  |  |  |  | 2420 | 2579 |
| Malta |  |  |  |  |  |  | 1614 | 1839 |
| Mauritius |  |  |  |  |  |  | 2299 | 2355 |
| Miranda-Venezuela |  |  |  |  |  |  | 1272 | 1629 |
| Moldova |  |  |  |  |  |  | 2658 | 2536 |
| Panama |  |  |  |  |  |  | 1936 | 2033 |
| Shanghai (China) |  |  |  |  |  |  | 2528 | 2587 |
| Singapore |  |  |  |  |  |  | 2626 | 2657 |
| Tamil Nadu (India) |  |  |  |  |  |  | 1720 | 1490 |
| Trinidad and Tobago |  |  |  |  |  |  | 2283 | 2495 |
| United Arab Emirates |  |  |  |  |  |  | 5554 | 5313 |

Table S5 notes:

1. The Liechtenstein sample was comparatively small.
2. According to PISA, the data from the Netherlands in 2000 did not fulfill the sampling criteria.
3. According to PISA, the data from the UK 2003 have sampling issues, but show reliable within-country gender differences.
4. The reading data of the USA in 2006 are missing and can therefore not be compared to the mathematics data.
5. For 2000, we report the sample size for the mathematics data set (the sample sizes of the reading data set are slightly different, due to the design of PISA 2000).
