# Supplementary material for: Sex Differences in Mathematics and Reading Achievement Are Inversely Related: Within- and Across-Nation Assessment of 10 Years of PISA Data
Source: PLoS One. 2013 Mar 13;8(3):e57988. doi: 10.1371/journal.pone.0057988 (PMC3596327; doi:10.1371/journal.pone.0057988)
Supplement: Table S6 — Human development and gender equality scores. HDI: Human Development Index. GII = Gender Inequality Index. GDI = Gender Development Index. GEM = Gender Empowerment Measure. GGGI = Global Gender Gap Index. Gini = Gini coeffient. We correlated the magnitude of the sex differences in mathematics and reading for each year with each of these variables to determine if a consistent pattern of correlation emerged (i.e., the sex differences in mathematics in each PISA assessment correlates with the variable). No such pattern was found (Table 3). (DOC) [file pone.0057988.s007.doc]

| **Country/Region** | **HDI** | **GII** | **GDI** | **GEM** | **GGGI** | **Gini** |
| --- | --- | --- | --- | --- | --- | --- |
| Australia | 0.96 | 0.13 | 0.96 | 0.87 | 0.72 | 35.19 |
| Austria | 0.95 | 0.14 | 0.93 | 0.75 | 0.72 | 29.15 |
| Belgium | 0.95 | 0.08 | 0.94 | 0.84 | 0.72 | 32.97 |
| Canada | 0.97 | 0.13 | 0.96 | 0.83 | 0.71 | 32.56 |
| Czech Republic | 0.90 | 0.15 | 0.89 | 0.65 | 0.68 | 25.82 |
| Denmark | 0.95 | 0.06 | 0.95 | 0.89 | 0.75 | 24.7 |
| Finland | 0.95 | 0.09 | 0.95 | 0.89 | 0.82 | 26.88 |
| France | 0.96 | 0.11 | 0.95 | 0.78 | 0.73 | 32.74 |
| Germany | 0.94 | 0.09 | 0.94 | 0.85 | 0.74 | 28.31 |
| Greece | 0.95 | 0.16 | 0.94 | 0.69 | 0.67 | 34.27 |
| Hungary | 0.88 | 0.24 | 0.88 | 0.59 | 0.69 | 31.18 |
| Iceland | 0.97 | 0.11 | 0.96 | 0.88 | 0.80 | - |
| Ireland | 0.96 | 0.18 | 0.94 | 0.73 | 0.75 | 34.28 |
| Italy | 0.94 | 0.11 | 0.94 | 0.73 | 0.68 | 36.03 |
| Japan | 0.96 | 0.13 | 0.94 | 0.57 | 0.64 | 24.85 |
| Korea | 0.93 | 0.17 | 0.92 | 0.54 | 0.62 | - |
| Luxembourg | 0.96 | 0.18 | 0.94 | - | 0.68 | 30.76 |
| Mexico | 0.84 | 0.45 | 0.84 | 0.60 | 0.64 | 48.28 |
| New Zealand | 0.94 | 0.17 | 0.94 | 0.82 | 0.79 | 36.17 |
| Norway | 0.97 | 0.08 | 0.96 | 0.92 | 0.82 | 25.79 |
| Poland | 0.88 | 0.16 | 0.87 | 0.62 | 0.70 | 34.07 |
| Portugal | 0.90 | 0.14 | 0.90 | 0.74 | 0.71 | 38.45 |
| Spain | 0.95 | 0.12 | 0.94 | 0.82 | 0.73 | 34.66 |
| Sweden | 0.96 | 0.06 | 0.96 | 0.92 | 0.81 | 25.00 |
| Switzerland | 0.96 | 0.08 | 0.95 | 0.83 | 0.74 | 33.68 |
| United Kingdom | 0.94 | 0.20 | 0.94 | 0.79 | 0.74 | 35.97 |
| United States | 0.95 | 0.29 | 0.94 | 0.77 | 0.72 | 40.81 |
| Albania | 0.81 | 0.31 | 0.80 | - | 0.66 | 34.51 |
| Argentina | 0.86 | 0.37 | 0.86 | 0.69 | 0.72 | 44.49 |
| Brazil | 0.81 | 0.45 | 0.80 | 0.50 | 0.67 | 54.69 |
| Bulgaria | 0.83 | 0.24 | 0.83 | 0.60 | 0.71 | 28.19 |
| Chile | 0.87 | 0.38 | 0.86 | 0.52 | 0.68 | 52.06 |
| Hong Kong | 0.94 | - | 0.94 | - | - | 43.44 |
| Indonesia | 0.73 | 0.52 | 0.72 | 0.44 | 0.65 | 34.01 |
| Israel | 0.93 | 0.17 | - | 0.66 | 0.69 | 39.20 |
| Latvia | 0.86 | 0.20 | 0.86 | 0.64 | 0.74 | 36.61 |
| Liechtenstein | - | - | - | - | - | - |
| Macedonia | 0.81 | - | 0.80 | 0.64 | 0.69 | - |
| Peru | 0.79 | 0.41 | 0.78 | 0.63 | 0.70 | 48.14 |
| Romania | 0.82 | 0.33 | 0.82 | 0.50 | 0.68 | 30.00 |
| Russia | 0.81 | 0.33 | 0.80 | 0.54 | 0.70 | - |
| Thailand | 0.79 | 0.38 | 0.78 | 0.51 | 0.69 | 40.02 |
| The Netherlands | 0.96 | 0.04 | 0.95 | 0.87 | 0.74 | 30.90 |
| Slovakia | 0.87 | 0.19 | 0.87 | 0.64 | 0.68 | 26.00 |
| Turkey | 0.80 | 0.44 | 0.78 | 0.37 | 0.59 | 38.95 |
| Macao | - | - | - | - | - | - |
| Serbia | - | - | - | - | - | 27.80 |
| Tunisia | 0.76 | 0.33 | 0.75 | - | 0.63 | 41.42 |
| Uruguay | 0.86 | 0.37 | 0.86 | 0.54 | 0.69 | 45.32 |
| Azerbaijan | 0.76 | - | 0.76 | 0.43 | 0.69 | 33.71 |
| Chinese Taipei | - | - | - | - | - | - |
| Colombia | 0.79 | 0.50 | 0.78 | 0.49 | 0.69 | 55.91 |
| Croatia | 0.86 | 0.18 | 0.86 | 0.62 | 0.7 | 33.65 |
| Estonia | 0.87 | 0.19 | 0.87 | 0.66 | 0.71 | 36.00 |
| Jordan | 0.77 | 0.48 | 0.76 | - | 0.63 | 35.43 |
| Kyrgyzstan | 0.69 | 0.36 | 0.69 | 0.57 | - | - |
| Lithuania | 0.87 | 0.21 | 0.87 | 0.61 | 0.72 | 37.57 |
| Montenegro | - | - | - | - | - | 29.99 |
| Qatar | - | 0.55 | - | - | 0.59 | 41.10 |
| Slovenia | 0.92 | 0.18 | 0.92 | 0.62 | 0.69 | 31.15 |
| Georgia | 0.76 | - | - | 0.40 | 0.67 | 41.34 |
| Costa Rica | 0.85 | 0.37 | 0.84 | 0.69 | 0.71 | 50.73 |
| Himachal Pradesh (India) | 0.61 | - | 0.59 | - | 0.61 | - |
| Kazakhstan | 0.81 | 0.34 | 0.80 | 0.52 | 0.70 | 29.04 |
| Malaysia | 0.82 | 0.28 | 0.82 | 0.54 | 0.64 | 46.21 |
| Malta | 0.89 | 0.24 | 0.89 | 0.53 | 0.66 | - |
| Mauritius | 0.80 | 0.37 | 0.80 | 0.51 | 0.65 | - |
| Miranda (Venezuela) | 0.83 | - | 0.82 | 0.58 | 0.69 | - |
| Moldova | 0.72 | 0.29 | 0.72 | 0.55 | 0.72 | 33.03 |
| Panama | 0.83 | 0.46 | 0.83 | 0.60 | 0.71 | 51.92 |
| Shanghai-China | 0.76 | - | 0.76 | 0.53 | 0.69 | - |
| Singapore | 0.92 | 0.08 | 0.9 | 0.78 | 0.66 | 42.48 |
| Tamil Nadu (India) | 0.61 | - | 0.59 | - | 0.61 | - |
| Trinidad and Tobago | 0.83 | 0.32 | 0.83 | 0.78 | 0.72 | 40.27 |
| United Arab Emirates | 0.90 | 0.20 | 0.88 | 0.70 | 0.62 | - |
